# Bioactive compounds from *Tetradenia riparia* and *Tetracera poggei*: antioxidant activity, UPLC-QTOF-MS phytochemical profiling, and *in silico* targeting of the RNA-binding protein DRBD18 of *Trypanosoma brucei*

**DOI:** 10.3389/fphar.2026.1910102

**Published:** 2026-09-22

**Authors:** Williams Balela Balela, Olivier Mpungi Konde, Sephora Mutombo Mianda, Erdi Can Aytar, Jean-Paul Ngbolua Koto-te-Nyiwa, Joseph Iteku Bekomo, Tania Bishola Tshitenge

**Affiliations:** 1 Department of Life Sciences, Faculty of Sciences and Technology, University of Kinshasa, Kinshasa, Democratic Republic of Congo; 2 Laboratory of Ethnobiology and Medical Phytochemistry (E-PHYMED), University of Kinshasa, Kinshasa, Democratic Republic of Congo; 3 Laboratory for Analysis and Research on Food and Nutrition (LARAN), University of Kinshasa, Kinshasa, Democratic Republic of Congo; 4 Department of Medicinal Chemistry and Pharmacognosy, Faculty of Pharmaceutical Sciences, University of Kinshasa, Kinshasa, Democratic Republic of Congo; 5 Department of Horticulture, Faculty of Agriculture, Usak University, Usak, Türkiye; 6 One Health Institute for Africa (INOHA), University of Kinshasa, Kinshasa, Democratic Republic of Congo; 7 Department of Epidemiology and Global Health, Institut National de Recherche Biomédicale (INRB), Kinshasa, Democratic Republic of Congo

**Keywords:** ABTS, antioxidant activity, DPPH, DRBD18, molecular docking, phytochemistry, Tetracera poggei, Tetradenia riparia

## Abstract

Oxidative stress has been implicated in the pathogenesis of African trypanosomiasis. Therefore, plant-derived antioxidants have been investigated as potential therapeutic agents, as they may not only reduce oxidative damage by scavenging reactive oxygen species but also impair parasite survival. The present study investigates the antioxidant activity and phytochemical composition of two Central African medicinal plants, *Tetradenia riparia* (Hochst.) Codd and *Tetracera poggei* (Gilg.), and evaluates the potential binding of their major constituents to the mRNA-binding protein DRBD18 of *Trypanosoma brucei* through *in silico* molecular docking. Aqueous and methanolic extracts were prepared by decoction and percolation. Qualitative phytochemical screening and quantitative spectrophotometry (Folin–Ciocalteu, AlCl_3_) revealed rich profiles of polyphenols, flavonoids, tannins, anthocyanins, and alkaloids in both species. UPLC-QTOF-MS in negative ESI mode identified eleven compounds in *T. riparia*, including rosmarinic acid, luteolin, acacetin, and diterpenoid SP-II and thirteen compounds in *T. poggei*, dominated by galloylated catechin derivatives (epi-gallocatechin 3-O-gallate, catechin 3-O-gallate (−)-epicatechin-3-(3-O-methyl)-gallate). Antioxidant activity by DPPH and ABTS radical scavenging assays showed that percolated extracts consistently outperformed decocted extracts; *T. poggei* percolated extract showed the strongest DPPH activity (IC_50_ = 4.141 ± 0.175 μg/mL). Molecular docking against the RRM1 and RRM2 domains of DRBD18 (AlphaFold model AF-Q57XR9-F1) revealed that diterpenoid SP-II and (−)-epicatechin-3-(3-O-methyl)-gallate displayed the highest binding affinities (RRM1: −7.3 kcal/mol). The strong antioxidant capacity of the identified compounds, particularly galloylated catechins and rosmarinic acid, is routinely linked to their structural features that also favor RRM domain binding, highlighting their potential as antioxidant compounds and candidate DRBD18 ligands warranting further biological investigation. These findings provide a rational basis for the further development of plant-derived agents targeting oxidative stress and RNA-binding mechanisms in *T. brucei*.

## 1 Introduction

African Trypanosomiasis is a neglected tropical disease caused by protozoan flagellates of the genus *Trypanosoma*, transmitted by tsetse flies (*Glossina* spp.) across sub-Saharan Africa (Venturelli et al., 2022). Human African trypanosomiasis (HAT) is caused by *Trypanosoma brucei gambiense* in West and Central Africa and by *T b. rhodesiense* in East and Southern Africa, while Animal African trypanosomiasis (AAT) is caused by *T b. brucei*, *T. congolense*, and *T. vivax* (Venturelli et al., 2022). AAT alone threatens more than 55 million cattle and imposes critical barriers to food security and economic development (Cecchi et al., 2025). The Democratic Republic of the Congo (DRC) remains one of the most affected endemic countries (Hasker et al., 2012).

A well-established but underappreciated aspect of trypanosomiasis pathogenesis is the induction of oxidative stress in the mammalian host. *Trypanosoma brucei* infection triggers a strong inflammatory response associated with excessive production of reactive oxygen species (ROS), including superoxide anions, hydrogen peroxide, and hydroxyl radicals, by activated macrophages and neutrophils at the site of infection (Kasozi et al., 2022). The excessive production of reactive oxygen species can exceed the host’s antioxidant defense system, resulting in lipid peroxidation, DNA damage, and tissue injury. These changes have been linked to the neurological complications observed in late-stage HAT (Vincendeau and Bouteille, 2006). In contrast, trypanosomes depend on a unique thiol-based antioxidant system, with trypanothione playing a central role in protecting the parasite against oxidative stress arising from both its own metabolism and the host immune response. Because the parasite relies heavily on this defense mechanism for survival, interference with the trypanosome system can be fatal, making redox pathways an attractive target for the development of new antitrypanosomal therapies (Fairlamb and Horn, 2018). Plant-derived antioxidants therefore hold dual promise; they may attenuate host oxidative damage while simultaneously interfering with parasite antioxidant defense or other essential biological functions.

Currently available antitrypanosomal drugs including pentamidine, suramin, melarsoprol, eflornithine, and fexinidazole are associated with significant limitations such as complex parenteral administration, severe toxic side effects, emerging drug resistance, and high costs inaccessible to the most vulnerable populations (Hotez et al., 2014; Pereira et al., 2024). These constraints highlight the urgent need for novel, safe, and affordable therapeutic agents derived from renewable natural sources.

At the molecular level, trypanosomes regulate gene expression almost exclusively by post-transcriptional mechanisms. RNA-binding proteins (RBPs) are central regulators of mRNA stability, *trans*-splicing, polyadenylation, and translation (Clayton, 2014; Clayton, 2019). DRBD18 (Double RRM-containing protein 18; UniProt ID: Q57XR9) is an essential *T. brucei* RBP that regulates mRNA *trans*-splicing and polyadenylation of transcripts containing polypyrimidine tracts in their 3′-UTR (Tshitenge and Clayton, 2022). DRBD18 has also been identified as a transcript-specific RNA editing auxiliary factor (Pandey et al., 2025). Its depletion by RNA interference severely impairs parasite growth, making it a compelling drug target. Polyphenolic compounds, known for their strong antioxidant properties, have been proposed as candidates capable of disrupting RRM domain–RNA interactions through hydrogen bonding and aromatic stacking (Xu and Wang, 2025; Lešnik et al., 2025).

In this study, two Central African medicinal plants were selected, *Tetradenia riparia* (Hochst.) Codd (Lamiaceae), widely used for its insecticidal, antiparasitic, antimalarial, anti-inflammatory, antibacterial, antifungal, antidiarrheal, antitumor and analgesic properties (Panda et al., 2022; Sena et al., 2024) and *Tetracera poggei* Gilg (Dilleniaceae), a lesser-studied species whose leaves are traditionally used for infectious diseases in Central Africa, with no prior UPLC-MS or computational study reported (Ogunlakin and Sonibare, 2022). Previous work from our group identified rosmarinic acid as a major compound of *T. riparia* (Konde et al., 2025; Ngolo et al., 2025).

The present study aims to (i) characterize the phytochemical composition of *T. riparia* and *T. poggei* by TLC screening, quantitative spectrophotometry, and UPLC-QTOF-MS; (ii) evaluate the antioxidant activity of decocted and percolated extracts by DPPH and ABTS assays; and (iii) perform *in silico* molecular docking of the major identified compounds against DRBD18 RRM1 and RRM2 domains to identify potential DRBD18 ligands and candidate inhibitors warranting further biological investigation.

## 2 Materials and methods

### 2.1 Plant collection and preparation

Leaves of *T. riparia* were collected between March and April 2025 in the Ngansele district, Mont-Ngafula commune, Kinshasa, DRC (S 4°26’17.197”; E 15°17’32.812”; altitude 0 m) while leaves of *T. poggei* (local names: Bojo, Monji) were collected in April 2025 in the Kimwenza area, Mont-Ngafula commune (S 4°27’4.251”; E 15°17′37.693″). Collections were carried out during the period when healthy, fully expanded mature leaves were readily available for sampling. Plant collection adhered to all ethical guidelines; permission was obtained from the Faculty of Sciences and Technologies, University of Kinshasa. Voucher specimens (3,970 for *T. riparia* and 2,745 for *T. poggei*) were deposited at the Herbarium of Kinshasa (IUK), INERA-RDC. Collected plant material was oven-dried (Raypa) at 40 °C for 4 days and ground using an electric grinder (Blinder Butterfly B-592). Geographic collection sites are shown in Figure 1.

**FIGURE 1:**
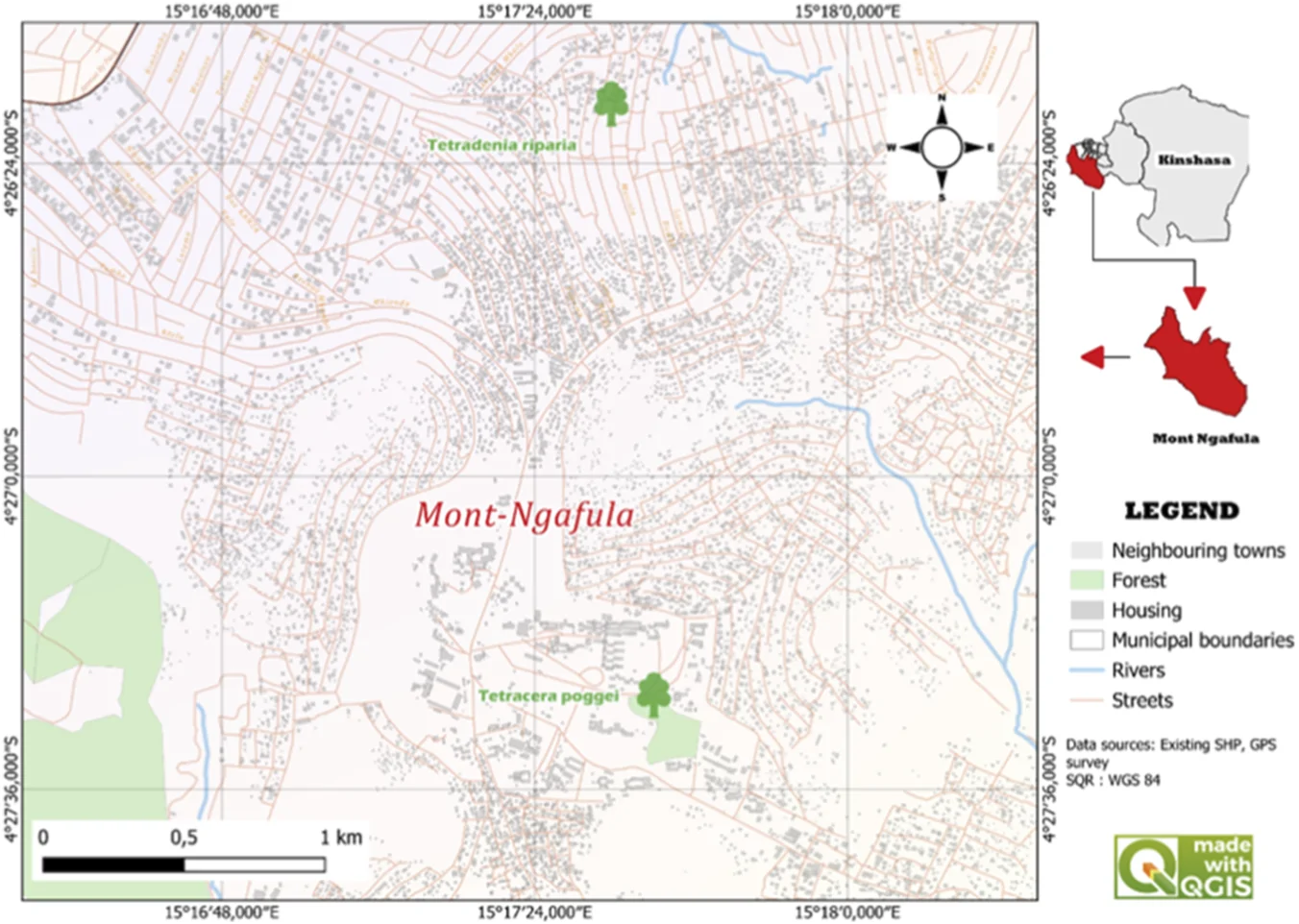
Geographical location of the collection sites for the leaves of *Tetradenia riparia* and *Tetracera poggei*.

### 2.2 Preparation of extracts

Extracts were prepared by decoction and percolation following the protocol of Ngolo et al. (2025), with minor modifications. For decoction, 10 g of powder were mixed with 100 mL of distilled water, heated at 100 °C for 10 min, cooled, and filtered through Whatman filter paper. For percolation, 10 g of powder were macerated cold in 50 mL of dichloromethane/methanol (1:1, v/v) for 48 h and the percolate collected dropwise. Both extracts were evaporated and stored as dry extracts at 4 °C. Extraction yield (R) was calculated as:

R (%) = (Mex/Mmv) × 100, where Mex is the dry residue mass and Mmv is the initial dry plant material mass.

### 2.3 Phytochemical screening

Qualitative phytochemical screening was performed on the aqueous and organic extracts following the methods of Hamilton-Amachree et al. (2024); Shaikh and Patil (2020), and Tourabi et al. (2025) detecting phenols, flavonoids, alkaloids, saponins, quinones, terpenoids, anthraquinones, tannins, anthocyanins, leucoanthocyanins, and steroids. Total polyphenol content was determined by the Folin–Ciocalteu method with gallic acid as standard. Total flavonoid content was quantified by the AlCl_3_ colorimetric method (Mbadiko et al., 2019). All measurements were performed in triplicate, and results are expressed as mean ± SD.

### 2.4 UPLC-QTOF-MS analysis

Compound identification was carried out following the protocol of Ngolo et al. (2025). Dry extract (1 mg) was re-dissolved in 1 mL MeOH/water (1:1) and filtered through a 0.22 µm nylon syringe filter. Analysis was performed on a Waters Acquity UPLC system (Waters BEH C18 column, 2.1 × 100 mm, 1.7 µm) coupled to a Waters Synapt G2 QTOF mass spectrometer in negative ESI mode, as described previously (Ngolo et al., 2025). Chromatographic separation used a gradient of solvent A (water + 0.1% formic acid) and solvent B (methanol + 0.1% formic acid): 0 min, 3% B; 0.10 min, 3% B; 14 min, 100% B; 16 min, 100% B; 16.5 min, 3% B; 20 min, 3% B. Flow rate: 0.3 mL/min; injection volume: 5 µL. ESI source parameters (negative mode) were as follows; source temperature 120 °C; sampling cone 20 V; extraction cone 4.0 V; desolvation temperature 300 °C; cone gas 10.0 L/h; desolvation gas 600 L/h; capillary 2.6 kV; trap collision energy 28 V. Molecular formulae were generated with MassLynx V4, and tentative identifications were made by comparing fragmentation patterns with the KEGG and PubChem databases via MetFrag (https://msbi.ipb-halle.de/MetFrag/).

### 2.5 Antioxidant activity

Radical scavenging activity was evaluated by DPPH (2,2-diphenyl-1-picrylhydrazyl) and ABTS (2.2′-azino-bis(3-ethylbenzothiazoline-6-sulphonic acid)) assays as described by Bukatuka et al. (2016) with minor modifications. For DPPH, 1980 µL of 0.1 mM DPPH in methanol was mixed with 20 µL of extract at varying concentrations, incubated in the dark for 30 min, and absorbance read at 517 nm. For ABTS, the radical cation was generated by reacting 20 mM ABTS with 10 mM K_2_S_2_O_8_ (1:1) for 16 h in the dark; the working solution was diluted to an absorbance of 0.8–1.0, mixed with extract, and read at 734 nm after 30 min. IC_50_ values were calculated from inhibition–concentration curves using GraphPad Prism 9.5.1.733. All measurements were performed in triplicate.

### 2.6 Molecular docking studies

#### 2.6.1 Protein preparation

*Trypanosoma brucei* DRBD18 (UniProt: Q57XR9; gene: Tb927.11.14090) was selected as the target protein (Pandey et al., 2025). The AlphaFold-predicted model (AF-Q57XR9-F1, version 6) was retrieved from the AlphaFold Protein Structure Database (Jumper et al., 2021) and visualized in PyMOL (v2.5) and NGL Viewer. The model comprises 480 residues with two RRM domains: RRM1 (residues 90–170) and RRM2 (residues 260–340). Low-confidence terminal regions (low pLDDT scores) were excluded; water molecules and non-standard atoms were removed. Binding pocket coordinates were defined within the previously characterized RRM1 and RRM2 RNA-binding domains of DRBD18: RRM1 (x = 3, y = 12, z = −25) and RRM2 (x = −34, y = 3, z = 24).

#### 2.6.2 Ligand selection and docking

Ligands were selected from UPLC-MS profiles based on peak abundance and structural diversity. For *T. riparia*, rosmarinic acid, astragalin, luteolin, acacetin, asiatic acid, diterpenoid SP-II, abieta-8,11,13-triene-7,15,18-triol, and trihydroxy octadecenoic acid were putatively identified and used. For *T. poggei*, epi-gallocatechin 3-O-gallate, catechin 3-O-gallate (−)-epicatechin-3-(3-O-methyl)-gallate, isorhamnetin, 7-O-methylquercetin, 7-O-methylkaempferol, galloyl-hexose-sulfate, and 1-O-palmitoyl-3-O-(6-sulfo-α-D-quinovopyranosyl)-glycerol were also identified and used. Three-dimensional structures were retrieved from PubChem and prepared using Open Babel v3.1.0 prior to docking. During ligand preparation, hydrogen atoms and atom types required for docking were assigned automatically. Molecular docking was performed with AutoDock Vina v1.2.5 implemented in PyRx 0.9.8 (Trott and Olson, 2010). During docking, the receptor was treated as rigid, whereas all ligands were treated as flexible using the default AutoDock Vina protocol. The lowest binding energy (ΔG) pose was retained for further analysis. Protein–ligand interactions were visualized using BIOVIA Discovery Studio Visualizer.

## 3 Results

### 3.1 Extraction yields

The extraction yields of the aqueous (decoction) and methanolic (percolation) extracts are presented in Table 1. The aqueous and methanolic extracts of *Tetradenia riparia* yielded 11.8% and 11.9% respectively, indicating nearly identical extraction efficiencies. For *Tetracera poggei*, the aqueous and methanolic extracts yielded 11.6% and 21.7%, respectively. Unlike *T. riparia*, the methanolic extracts of *T. poggei* showed an extraction yield that was almost twice that of the aqueous extract.

**TABLE 1:** Extraction yield of the different plant extracts.

Plant extracts	Extraction yield of the plant extracts (%)
Decoction of *T. riparia*	11.8
Percolate of *T. riparia*	11.9
Decoction of *T. poggei*	11.6
Percolate of *T. poggei*	21.7

### 3.2 Qualitative phytochemical screening

The results of the qualitative phytochemical screening are presented in Table 2, highlighting the presence or absence of the major classes of secondary metabolites in the aqueous and organic extracts of both plant species. Polyphenols, flavonoids, anthocyanins, bound quinones, alkaloids, leucoanthocyanins, and saponins were detected in the aqueous extracts of both species. In the organic fractions, steroids were present in both species, while triterpenoids were identified exclusively in *T. riparia* and free quinones exclusively in *T. poggei*. Tannins were absent from the aqueous extract of *T. poggei*.

**TABLE 2:** Qualitative phytochemical screening of aqueous and organic extracts of *T. riparia* and *T. poggei*. + indicates presence; − indicates absence.

Phytochemical compounds	*T. riparia* (aqueous extract)	*T. riparia* (organic extract)	*T. poggei* (aqueous extract)	*T. poggei* (organic extract)
Polyphenols	+	—	+	—
Flavonoids	+	—	+	—
Anthocyanins	+	—	+	—
Tannins	+	—	−	—
Bound quinones	+	—	+	—
Alkaloids	+	—	+	—
Leucoanthocyanins	+	—	+	—
Saponins	+	—	+	—
Steroids	—	+	—	+
Free quinones	—	−	—	+
Triterpenoids	—	+	—	−

### 3.3 Total phenolic and flavonoid content

*Tetracera poggei* exhibited higher polyphenol and flavonoid contents (polyphenols: 510.227 ± 74.608 mgGAE/gDW; flavonoids: 855.394 ± 5.292 mgQE/gDW) than *T. riparia* (polyphenols: 331.56 ± 89.22 mgGAE/gDW; flavonoids: 445.727 ± 1.8 mgQE/gDW) (Table 3).

**TABLE 3:** Total polyphenol and flavonoid contents of the methanolic extracts (mean ± SD, n = 3).

Species	Polyphenols (mgGAE/gDW)	Flavonoids (mgQE/gDW)
*T. riparia*	331.56 ± 89.22	445.727 ± 1.8
*T. poggei*	510.227 ± 74.608	855.394 ± 5.292

GAE: gallic acid equivalent; QE: quercetin equivalent; DW: dry weight.

### 3.4 UPLC-QTOF-MS metabolite profiling

UPLC-QTOF-MS in negative ESI mode tentatively identified eleven compounds in the methanolic extracts of *T. riparia* (Figure 2; Table 3) and thirteen compounds in the methanolic extracts of *T. poggei* (Figure 3; Table 4).

**FIGURE 2:**
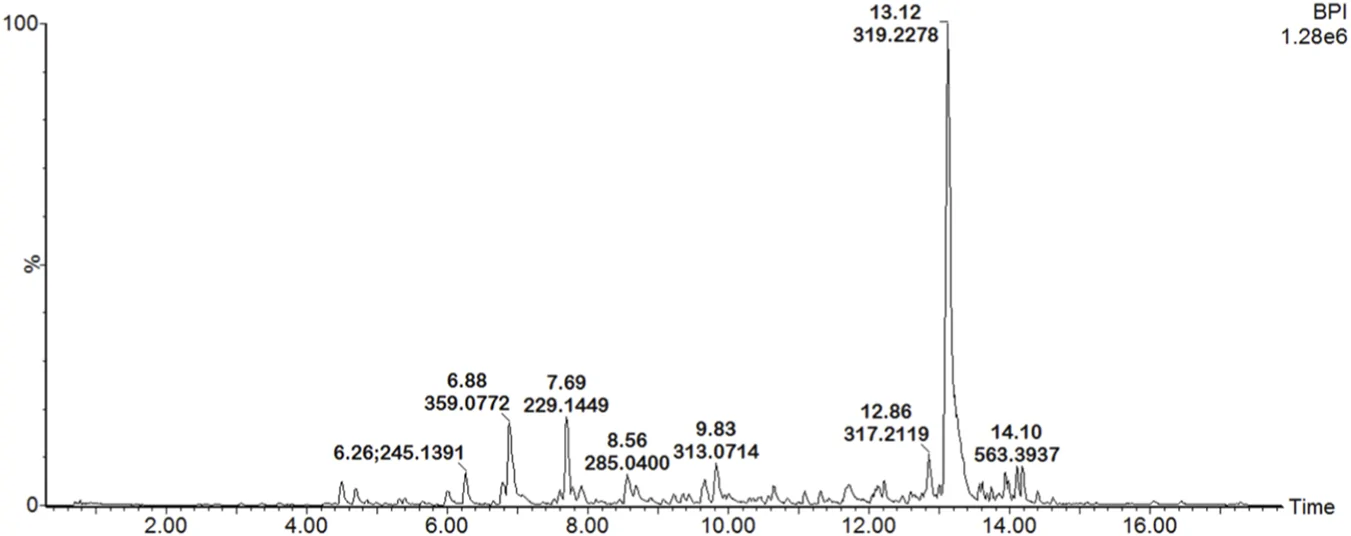
ESI negative mode BPI chromatogram of the *Tetradenia riparia* methanolic extract.

**FIGURE 3:**
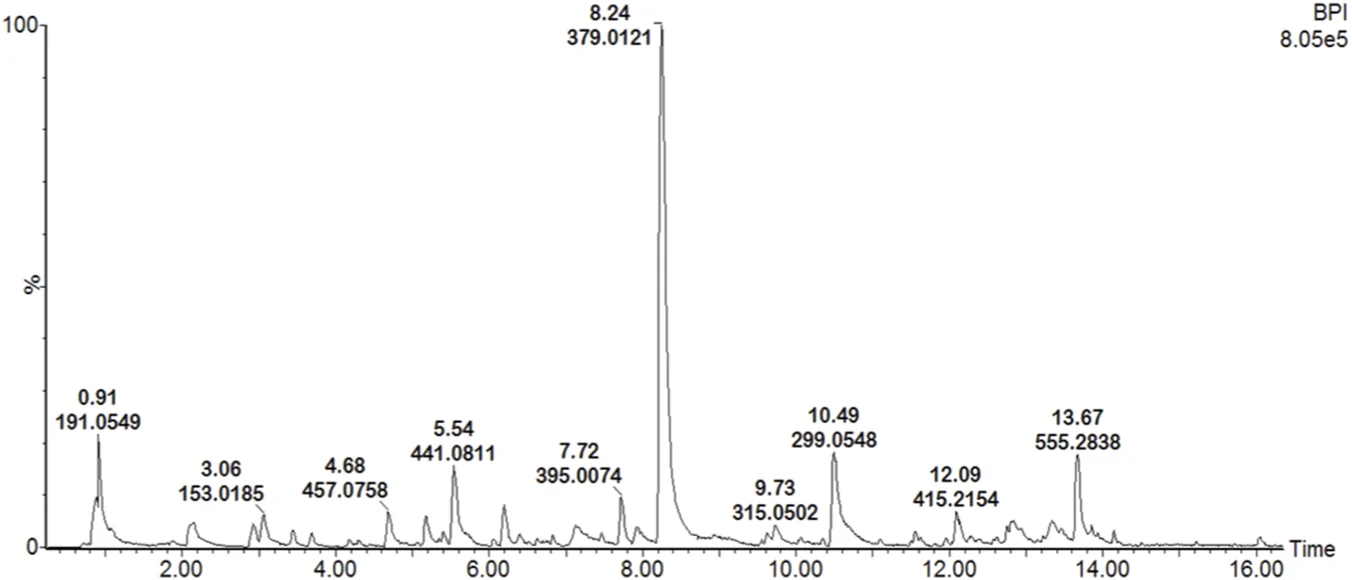
ESI negative mode BPI chromatogram of the *Tetracera poggei* methanolic extract.

**TABLE 4:** Tentative identification of the major compounds in the methanolic extracts from *Tetradenia riparia*.

RT (min)	Exp. m/z [M-H]^-^	Mol. formula	Theor. m/z [M-H]^-^	Error (ppm)	Compound
4.66	179.0337	C_9_H_8_O_4_	179.0344	−3.9	Caffeic acid
6.77	447.0926	C_21_H_20_O_11_	447.0927	−0.2	Astragalin
6.85	359.0772	C_18_H_16_O_8_	359.0767	1.4	Rosmarinic acid
7.66	229.1449	C_12_H_22_O_4_	229.1440	3.9	Dibutyl succinate
8.53	285.0400	C_15_H_10_O_6_	285.0399	0.4	Luteolin
9.61	327.2168	C_18_H_32_O_5_	327.2171	−0.9	Trihydroxy octadecenoic acid
9.78	313.0707	C_17_H_14_O_6_	313.0712	−1.6	Nepetoidin a or isomer
10.60	283.0617	C_16_H_12_O_5_	283.0606	3.9	Acacetin
11.66	487.3438	C_30_H_48_O_5_	487.3423	3.1	Asiatic acid
12.18	335.2220	C_20_H_32_O_4_	335.2222	−0.6	Diterpenoid SP-II
12.82	317.2119	C_20_H_30_O_3_	317.2117	0.6	Abieta-8,11,13-triene-7,15,18-triol

In *Tetradenia riparia* methanolic extracts (Figure 2), rosmarinic acid (m/z 359.0772, RT 6.85 min, error 1.4 ppm) was the dominant polar constituent, consistent with Ngolo et al. (2025) (Hamilton-Amachree et al., 2024). Caffeic acid (m/z 179.0337) and its biosynthetic precursor were also detected. Among the metabolites detected by UPLC-QTOF-MS, the flavonoids tentatively identified were astragalin (m/z 447.0926), luteolin (m/z 285.0400), acacetin (m/z 283.0617), and nepetoidin A or isomer (m/z 313.0707). Terpenoids included asiatic acid (m/z 487.3438), diterpenoid SP-II (m/z 335.2220), and abieta-8,11,13-triene-7,15,18-triol (m/z 317.2119). Trihydroxy octadecenoic acid (m/z 327.2168) and dibutyl succinate (m/z 229.1449) were also identified. The compounds tentatively identified are listed in Table 4.

In *Tetracera poggei* (Figure 3), the profile is dominated by galloylated catechin derivatives including epi-gallocatechin 3-O-gallate (m/z 457.0765, error −1.3 ppm), catechin 3-O-gallate (m/z 441.0818), and (−)-epicatechin-3-(3-O-methyl)-gallate (m/z 455.0967), alongside simpler phenolic acids such as gallic acid (m/z 169.0134), protocatechuic acid (m/z 153.0184), and methyl 6-O-galloyl-β-D-glucopyranoside (m/z 345.0818).

Methylated flavonols included isorhamnetin or isomer (m/z 315.0512), 7-O-methylquercetin or isomer (m/z 315.0498), and 7-O-methylkaempferol (m/z 299.0554). Sulphated compounds galloyl-hexose-sulfate (m/z 415.2162) and 1-O-palmitoyl-3-O-(6-sulfo-α-D-quinovopyranosyl)-glycerol (m/z 555.2838) were detected at later retention times. This chemical profile has not previously been reported for *T. poggei* and represents a significant expansion of the known phytochemistry of the genus *Tetracera* (Ogunlakin and Sonibare, 2022). The compounds tentatively identified are listed in Table 5.

**TABLE 5:** Tentative identification of the major compounds in the methanolic extracts from *Tetracera poggei*.

RT (min)	Exp. m/z [M-H]^-^	Mol. formula	Theor. m/z [M-H]^-^	Error (ppm)	Compound
0.90	191.0547	C_7_H_12_O_6_	191.0556	−4.7	Quinic acid
2.08	169.0134	C_7_H_6_O_5_	169.0137	−1.8	Gallic acid
2.90	345.0818	C_14_H_18_O_10_	345.0822	−1.2	Methyl 6-O-galloyl-β-D-glucopyranoside
3.05	153.0184	C_7_H_6_O_4_	153.0188	−2.6	Protocatechuic acid
4.66	457.0765	C_22_H_18_O_11_	457.0771	−1.3	Epi-gallocatechin 3-O-gallate (EGCG)
5.50	441.0818	C_22_H_18_O_10_	441.0822	−0.9	Catechin 3-O-gallate
6.17	455.0967	C_23_H_20_O_10_	455.0978	−2.4	(−)-Epicatechin-3-(3-O-methyl)-gallate
7.70	315.0512	C_16_H_12_O_7_	315.0505	2.2	Isorhamnetin or isomer
8.21	379.0121	C_16_H_12_O_9_S	379.0124	−0.8	Rhamnocitrin 3-O-sulfate
9.73	315.0498	C_16_H_12_O_7_	315.0505	−2.2	7-O-Methylquercetin or isomer
10.47	299.0554	C_16_H_12_O_6_	299.0556	−0.7	7-O-Methylkaempferol
12.08	415.2162	C_21_H_36_O_6_S	415.2154	1.9	Galloyl-hexose-sulfate
13.64	555.2838	C_25_H_48_O_11_S	555.2839	−0.2	1-O-Palmitoyl-3-O-(6-sulfo-α-D-quinovopyranosyl)-glycerol

### 3.5 Antioxidant activity

The antioxidant activities of decocted and percolated extracts are summarized in Table 6. Percolated extracts consistently showed higher radical scavenging activity than decocted extracts in both species. For DPPH, *T. poggei* percolated extract showed the strongest activity (IC_50_ = 4.141 ± 0.175 μg/mL), followed by *T. riparia* percolated extract (7.211 ± 0.222 μg/mL). In the ABTS assay, *T. riparia* percolated extract showed higher activity (IC_50_ = 10.79 ± 0.56 μg/mL) than *T. poggei* (13.99 ± 0.15 μg/mL). All IC_50_ values were below 100 μg/mL. Gallic acid was used as a positive control for the antioxidant activity assays.

**TABLE 6:** Antioxidant activities (IC_50_, µg/mL) of decocted and percolated extracts by DPPH and ABTS assays (mean ± SD, n = 3).

Species	DPPH Decoction	DPPH Percolation	ABTS Decoction	ABTS Percolation
*T. riparia*	14.826 ± 1.307	7.211 ± 0.222	18.14 ± 3.62	10.79 ± 0.56
*T. poggei*	8.713 ± 0.327	4.141 ± 0.175	7.044 ± 0.338	13.99 ± 0.15
*Gallic acid*	0.85 ± 0.15		0.75 ± 0.06	

### 3.6 Molecular docking against DRBD18

#### 3.6.1 *T. poggei* compounds

Table 7 summarizes the molecular docking results for the *T. poggei* compounds. Among the eight compounds evaluated (−)-epicatechin-3-(3-O-methyl)-gallate exhibited the most favorable predicted binding energy toward the RRM1 domain (ΔG = −7.3 kcal/mol), followed by epi-gallocatechin 3-O-gallate (ΔG = −7.2 kcal/mol) and catechin 3-O-gallate (ΔG = −7.1 kcal/mol). For the RRM2 domain, catechin 3-O-gallate showed the most favorable predicted binding energy (ΔG = −6.7 kcal/mol). Methylated flavonols exhibited moderate predicted binding energies (−5.7 to −6.6 kcal/mol for RRM1), whereas the sulphated lipid derivatives displayed the least favorable binding energies (−4.5 to −6.1 kcal/mol).

**TABLE 7:** Molecular docking results for *T. poggei* compounds against DRBD18 RRM1 and RRM2 domains.

Compound	Domain	ΔG (kcal/mol)
Epi-gallocatechin 3-O-gallate	RRM1	−7.2
​	RRM2	−5.9
Catechin 3-O-gallate	RRM1	−7.1
​	RRM2	−6.7
(−)-Epicatechin-3-(3-O-methyl)-gallate	RRM1	−7.3
​	RRM2	−5.9
Isorhamnetin	RRM1	−6.1
​	RRM2	−5.7
7-O-Methylquercetin	RRM1	−6.6
​	RRM2	−5.8
7-O-Methylkaempferol	RRM1	−6.2
​	RRM2	−5.7
Galloyl-hexose-sulfate	RRM1	−6.1
​	RRM2	−5.2
1-O-Palmitoyl-3-O-(6-sulfo-α-D-quinovopyranosyl)-glycerol	RRM1	−4.8
​	RRM2	−4.5

The interaction profiles of epi-gallocatechin-3-O-gallate (EGCG) at RRM1 and catechin-3-O-gallate (C3G) at RRM2 reveal distinct but well-defined binding characteristics (Supplementary Figure S1). Within the RRM1 binding pocket, EGCG forms conventional hydrogen bonds with residues GLY186, TYR177, ARG180, THR182, and GLU185, along with a π-sigma interaction with GLU181, π-alkyl contacts with LEU179 and ARG178, and a T-shaped π–π interaction with PHE188 (Figure 4). This pattern highlights the combined role of hydrogen bonding and aromatic contacts in RRM1 binding.

**FIGURE 4:**
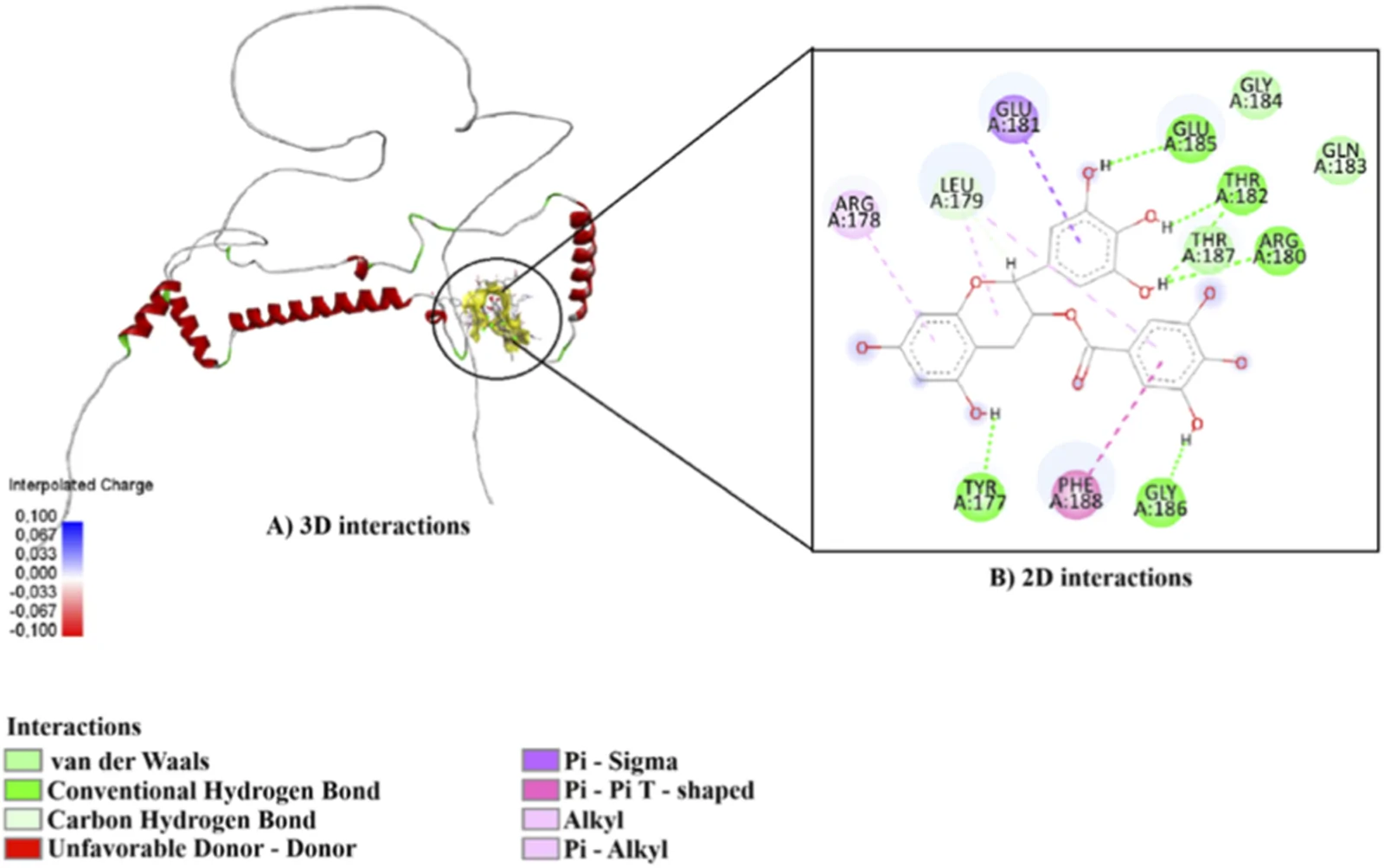
Molecular docking interaction profile of epi-gallocatechin-3-O-gallate with RRM1, showing **(A)** the 3D binding conformation in the active site and **(B)** a detailed 2D interaction map highlighting hydrogen bonds, van der Waals forces, and π-related interactions with key amino acid residues.

In contrast, the C3G interaction network at RRM2 is more complex and diverse; the compound forms a carbon hydrogen bond with THR232, conventional hydrogen bonds with ALA230, GLN243, and GLU231, a π-cation interaction with ARG247, and π–π stacking with TRP233, supplemented by π-alkyl interactions with ARG244 and LEU240 (Figure 5). This combination of electrostatic, aromatic, and hydrophobic interactions suggests a more balanced and versatile binding mode at RRM2. Interaction distances generally fall in the range of approximately 1.6–4.8 Å, indicating favorable spatial positioning of the ligands within both binding pockets.

**FIGURE 5:**
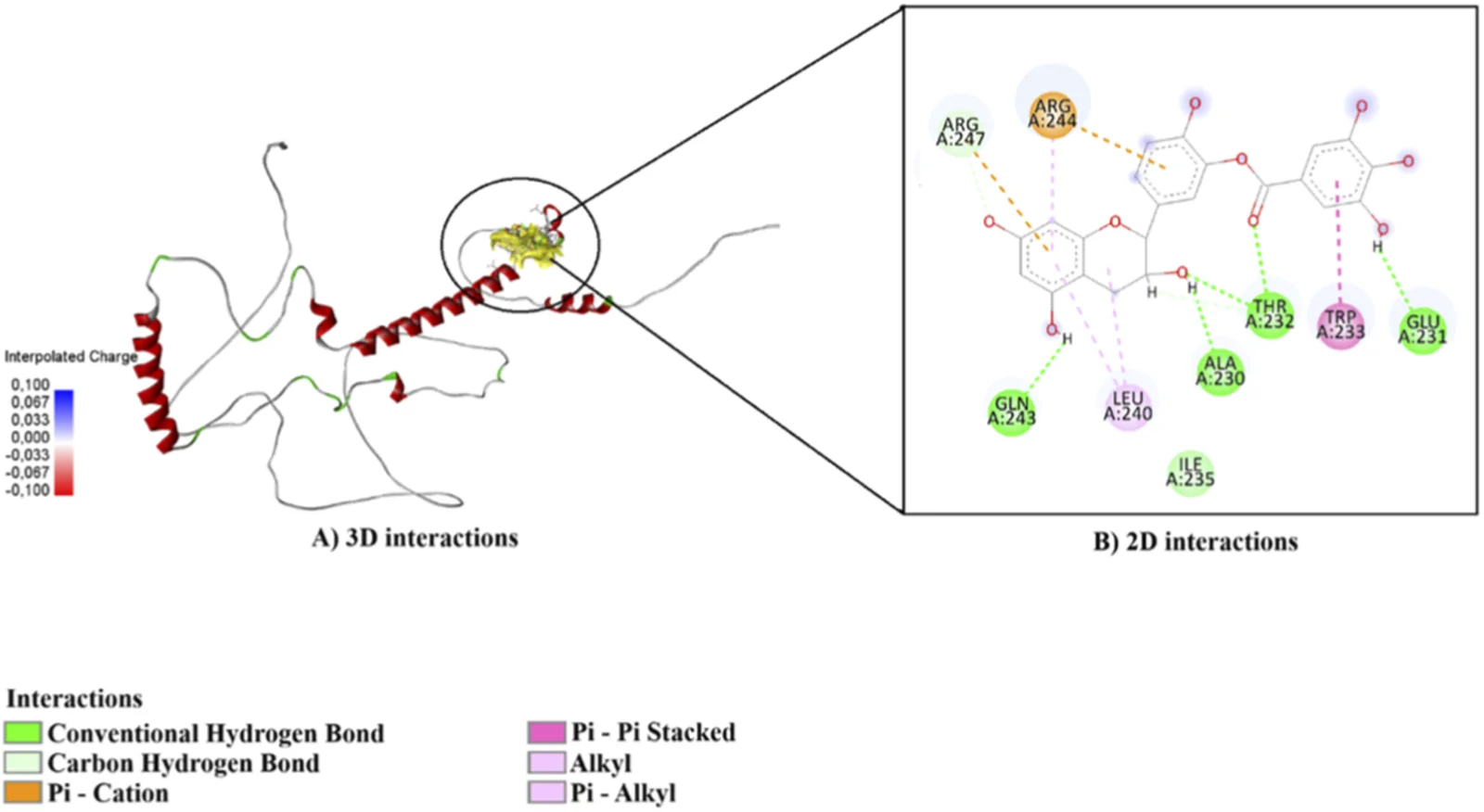
Molecular docking interaction profile of catechin-3-O-gallate with RRM2, illustrating **(A)** the 3D binding pose in the active site and **(B)** the 2D interaction diagram highlighting hydrogen bonds, π-cation, π–π stacking, and hydrophobic interactions with key amino acid residues.

#### 3.6.2 *T. riparia* compounds

Table 8 summarizes the molecular docking results for the *T. riparia* compounds. Diterpenoid SP-II exhibited the most favorable predicted binding energy toward the RRM1 domain (ΔG = −7.3 kcal/mol), followed by asiatic acid (ΔG = −7.2 kcal/mol), astragalin (ΔG = −6.8 kcal/mol), and abieta-8,11,13-triene-7,15,18-triol (ΔG = −6.8 kcal/mol). Phenylpropanoids and flavonoids, including rosmarinic acid (ΔG = −6.7 kcal/mol), luteolin (ΔG = −6.6 kcal/mol), and acacetin (ΔG = −6.5 kcal/mol), also exhibited favorable predicted binding energies toward the RRM1 domain. For the RRM2 domain, diterpenoid SP-II (ΔG = −6.1 kcal/mol) and acacetin (ΔG = −6.0 kcal/mol) showed the most favorable predicted binding energies. Trihydroxy octadecenoic acid exhibited the least favorable predicted binding energies (RRM1: ΔG = −3.5 kcal/mol; RRM2: ΔG = −2.9 kcal/mol).

**TABLE 8:** Molecular docking results for *T. riparia* compounds against DRBD18 RRM1 and RRM2 domains.

Compounds	Domain	ΔG (kcal/mol)
Rosmarinic acid	RRM1	−6.7
​	RRM2	−5.1
Astragalin	RRM1	−6.8
​	RRM2	−5.7
Luteolin	RRM1	−6.6
​	RRM2	−5.9
Acacetin	RRM1	−6.5
​	RRM2	−6.0
Asiatic acid	RRM1	−7.2
​	RRM2	−5.8
Abieta-8,11,13-triene-7,15,18-triol	RRM1	−6.8
​	RRM2	−5.7
Diterpenoid SP-II	RRM1	−7.3
​	RRM2	−6.1
Trihydroxy octadecenoic acid	RRM1	−3.5
​	RRM2	−2.9

The interaction profile of diterpenoid SP-II with RRM1 and RRM2 reveals limited but distinct contacts with both domains (Supplementary Figure S2). Within the RRM1 binding site, the compound forms an alkyl interaction with LEU179 and conventional hydrogen bonds involving GLY186 and THR182, reflecting structural complementarity between the diterpenoid scaffold and the RRM1 pocket (Figure 6).

**FIGURE 6:**
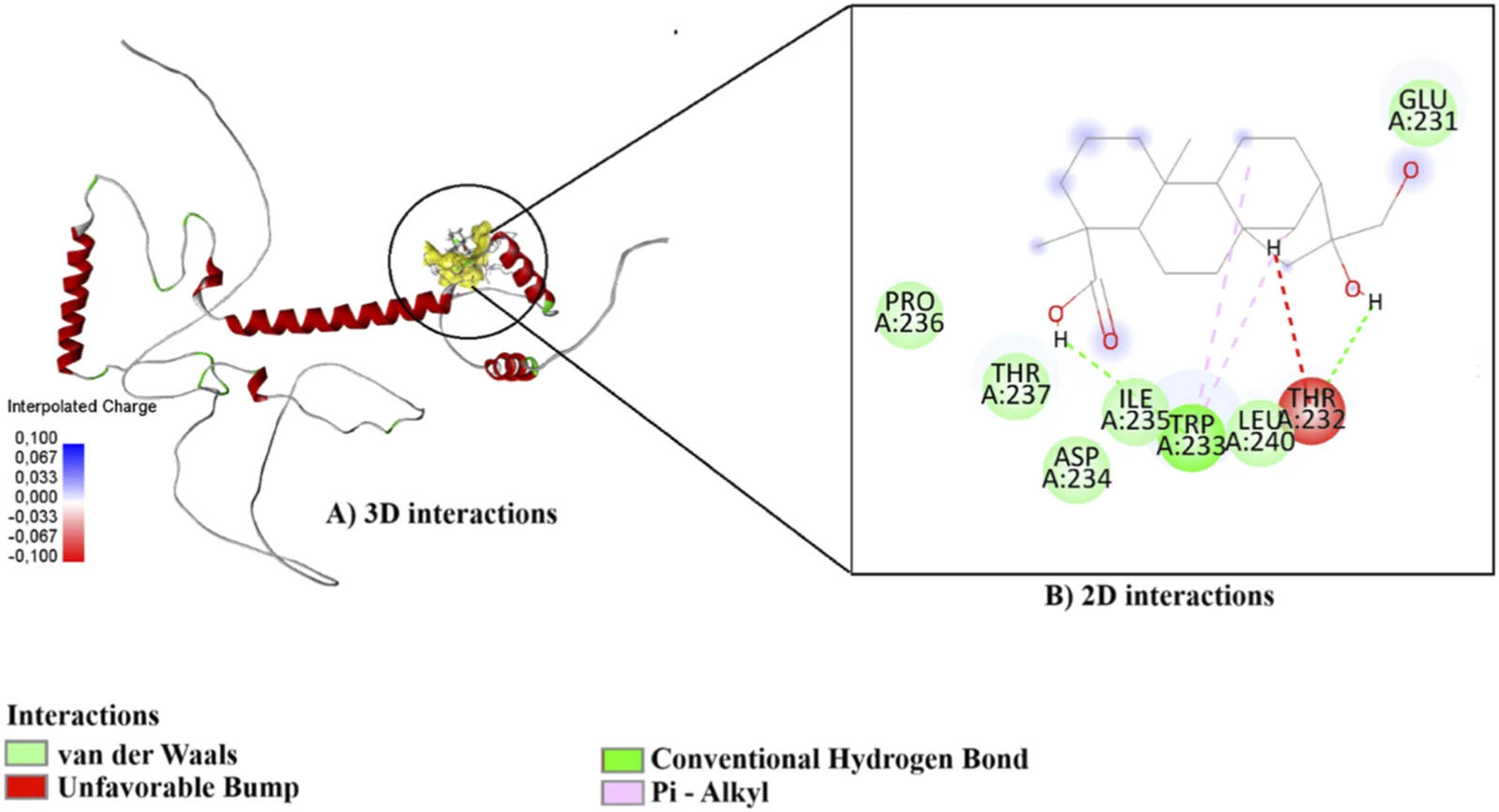
Molecular docking interaction profile of diterpenoid SP-II with RRM1, demonstrating **(A)** the 3D binding conformation in the active site and **(B)** the 2D interaction map highlighting hydrogen bonds, van der Waals forces, alkyl interactions, and donor–donor unfavorable contacts with key amino acid residues.

## 4 Discussion

The extraction yields obtained in the present study indicate that the extraction efficiency varied between *T. riparia* and *T. poggei.* For *T. riparia*, the aqueous and methanolic extracts exhibited nearly identical extraction yields (11.8% and 11.9%, respectively), suggesting that both solvents extracted comparable amounts of soluble constituents under the used extraction conditions. In contrast, the methanolic extract of *T. poggei* yielded almost twice as much extract as the aqueous extract (21.7% vs. 11.6%), indicating a greater recovery of compounds with intermediate polarity by methanol. These findings are consistent with the general observation that extraction yield depends on several factors, including solvent polarity, extraction method, solvent-to-plant material ratio, extraction time, geographical origin of the plant, environmental conditions and harvesting season (Panda et al., 2022). Methanol is widely recognized as an effective solvent for extracting a broad spectrum of phytochemicals, including phenolic compounds, flavonoids, and terpenoids whereas water preferentially extracts highly polar constituents such as carbohydrates, glycosides, tannins, and some phenolic compounds. Consequently, differences in solvent polarity can influence both the quantity and composition of recovered extracts (Lee et al., 2024).

The extraction yields obtained for *T. riparia* in the present study were higher than those reported by Ndamane et al. (2013), who obtained yields of 3.57% for the aqueous extract and 1.39% for the methanolic extract Ndamane et al. (2013). Such variations are expected and may reflect differences in several parameters, including the extraction method, plant material preparation, harvesting period, drying condition, environmental conditions, plant maturity; all of which have been shown to influence extraction efficiency and phytochemical recovery (Panda et al., 2022). The contrasting extraction patterns observed between *T. riparia* and *T. poggei* further suggest species-specific differences in phytochemical composition, with *T. poggei* potentially containing a higher proportion of methanol-soluble constituents than *T. riparia*.

The qualitative phytochemical screening demonstrated that both *T. riparia* and *T. poggei* contain several major classes of secondary metabolites, including flavonoids, polyphenols, tannins, and alkaloids. These metabolite classes have been widely associated with antioxidant, antimicrobial and antiparasitic activities, supporting the ethnomedicinal relevance of both species (Mutha et al., 2021; Caruso et al., 2022). A notable difference between the species was the detection of triterpenoids exclusively in *T. riparia* and free quinones exclusively in *T. poggei*, suggesting species-specific variation in secondary metabolite composition. The phytochemical profile of *T. riparia* obtained in this study is generally consistent with that reported by Hamilton-Amachree et al. (2024). However, unlike their findings, anthraquinones were not detected in this present study. The phytochemical profile of *T. poggei* observed in this study is generally consistent with previous reports on related medicinal plant species. For instance, Omotayo and Borokini (2012) reported the presence of several phytochemical constituents in the leaf extracts of *Tetracera potatoria*, except for steroids and cardiac glycosides (Omotayo and Borokini, 2012). Similarly, Nair et al. (2016) identified flavonoids, phenolics, tannins, saponins, and terpenoids in leaf extracts of *Tetracera akara* (Nair et al., 2016). In addition, Seudi et al. (2026) reported the presence of saponins, steroids, flavonoids, tannins, quinones, and coumarins in extracts prepared from the wild leaves of *Tetracera rosiflora* (Seudi et al., 2026). Although slight differences in the phytochemical profiles were observed among the studies, the occurrence of these major classes of secondary metabolites was generally comparable.

*Tetracera poggei* methanolic extracts exhibited 1.5-fold higher total polyphenol content and 1.9-fold higher total flavonoid content than *T. riparia* extracts. These values were higher than those reported by Chepng’etich et al. (2018) and Ngolo et al. (2025) for *T. riparia*, which may reflect differences in the harvest season, soil pH, drying conditions, geographic location, and extraction method (Daniels et al., 2010). The particularly high flavonoid content of *T. poggei* is consistent with the dense array of galloylated catechin derivatives subsequently identified by UPLC-MS. Phenolic compounds are known to modulate gene expression and exhibit antioxidant, antimutagenic, and antiparasitic activities (Xu and Wang, 2025). These activities are largely due to their hydroxyl groups, which scavenge free radicals and reduce oxidative stress within cells. Although the mechanisms underlying their antiparasitic effects are complex and vary among compounds, phenolics have been shown to interfere with parasite survival through multiple pathways, including disruption of membrane integrity and inhibition of key enzymatic processes. The identification of galloylated catechin derivatives in *T. poggei* by UPLC-QTOF-MS is therefore particularly relevant, because members of this class of compounds have previously been associated with antiparasitic activity in experimental studies (Argüello-Garcí and Quiñonez-Bastidas, 2020; Paveto et al., 2004).

UPLC-QTOF-MS analysis revealed complementary chemical profiles between the two species. In *T. riparia*, rosmarinic acid is the dominant polyphenol, already identified as a major constituent by Ngolo et al. (2025). Luteolin, acacetin, and astragalin are well-documented antioxidants. The terpenoid fraction containing diterpenoid SP-II, asiatic acid, and the abietane diterpene represent structurally diverse scaffolds not previously described by UPLC-MS in this species. In *T. poggei*, the dominance of EGCG, catechin 3-O-gallate, and (−)-epicatechin-3-(3-O-methyl)-gallate is noteworthy; the galloyl ester moiety of these compounds is a well-established structural determinant of both antioxidant capacity and protein-binding affinity (Saeki et al., 2018; Hirci et al., 2025). This chemical profile has not previously been reported for *T. poggei* (Ogunlakin and Sonibare, 2022).

Percolated extracts showed stronger antioxidant activity than decocted extracts in both the DPPH and ABTS assays. This difference is likely due to the greater extraction efficiency of the dichloromethane/methanol solvent system, which is more effective at recovering polyphenolic and terpenoid compounds than the aqueous decoction. The superior DPPH radical scavenging activity of *T. poggei* (IC_50_ = 4.141 μg/mL) is consistent with its significantly higher polyphenol and flavonoid contents and the presence of galloylated catechin derivatives identified by UPLC-QTOF-MS (Al-Mudhafar and Wadi, 2024). Although both DPPH and ABTS evaluate the antioxidant activity, they are based on different reaction mechanisms, which may explain the differences observed among the extracts. The DPPH assay mainly measures the ability of hydrophilic antioxidants to donate hydrogen atoms, whereas the ABTS assay detects the activity of both hydrophilic and lipophilic compounds. This broader detection may explain why *T. riparia* exhibited relatively stronger antioxidant activity in the ABTS assay, as its phytochemical profile includes lipophilic diterpenoids (Cano et al., 2023). The DPPH IC_50_ value of the percolated extract of *T. riparia* (7.211 ± 0.222 μg/mL) was lower than that reported by Ngolo et al. (2025). This difference may be attributed to variations in seasonal and geographic origin, environmental growing conditions, extraction procedures, or differences in the experimental conditions used for the DPPH assay. Phenolic compounds, including rosmarinic acid, gallic acid, caffeic acid, and the galloylated catechins are widely recognized for their contribution to antioxidant activity (Kavi et al., 2023). This is in agreement with the positive correlation observed in the present study between polyphenol and flavonoid contents and the IC_50_ values.

Importantly, many of the identified antioxidant compounds in both species are the same molecules that show the strongest binding affinities in the molecular docking analysis. This potential association between antioxidant activity and DRBD18 binding is systematically plausible; the structural features responsible for antioxidant activity, including multiple hydroxyl groups, aromatic rings, and electron-donating substituents, are also the features that favor hydrogen bonding and aromatic stacking interactions with RRM domain residues. EGCG and catechin 3-O-gallate are among the best-characterized RRM-binding polyphenols in the literature (Saeki et al., 2018; Hirci et al., 2025). In *T. poggei* (−)-epicatechin-3-(3-O-methyl)-gallate achieved the highest RRM1 binding energy (ΔG = −7.3 kcal/mol). Interaction analysis of EGCG at RRM1 reveals classical hydrogen bonds with GLY186, TYR177, ARG180, THR182, and GLU185, along with π-sigma (GLU181), π-alkyl (LEU179, ARG178), and T-shaped π–π (PHE188) interactions. The involvement of PHE188, a conserved aromatic residue in the RRM β-sheet that normally participates in RNA base stacking, strongly suggests direct competition with RNA substrates (Saeki et al., 2018; Hirci et al., 2025). At RRM2, catechin 3-O-gallate forms an extensive network including a π-cation interaction with ARG247 and π–π stacking with TRP233.

In *T. riparia*, diterpenoid SP-II (ΔG = −7.3 kcal/mol) and asiatic acid (−7.2 kcal/mol) achieved RRM1 affinities comparable to the best polyphenols, through a different mechanistic basis: primarily hydrophobic and van der Waals contacts rather than hydrogen bonding. Diterpenoid SP-II at RRM1 forms alkyl interactions with LEU179 and hydrogen bonds with GLY186 and THR182; at RRM2, it engages π-alkyl with TRP233 and a hydrogen bond with THR232. This terpenoid binding mode is consistent with previous reports that showed that natural diterpenoids can interact with RNA-binding proteins through hydrophobic surface complementarity. Although the polyphenols and diterpenoids interacted with DRBD18 in different ways, both classes produced similar RRM1 binding affinities. Taken together, these findings suggest that DRBD18 may accommodate chemically diverse ligands rather than only highly polar compounds.

The docking results presented here should be regarded as preliminary until they are confirmed experimentally. A key limitation of this study is that the analysis was performed using an AlphaFold-predicted DRBD18 structure, as no experimentally resolved crystal structure is currently available. In addition, molecular docking provides only a simplified and static representation of protein-ligand interactions and does not fully account for protein flexibility or solvent effects. The selectivity of the identified compounds towards mammalian homologues was also not evaluated. Furthermore, because no experimentally resolved DRBD18 structure with a co-crystallized ligand is currently available, conventional validation of the docking protocol through redocking or benchmarking against a reference ligand could not be performed. Future studies should therefore focus on experimental validation of these computational predictions through biochemical RNA-binding assays, evaluation of anti-trypanosomal activity in cultured parasites, assessment of selectivity against mammalian cells, and where possible, structural studies to confirm the predicted binding interactions.

## 5 Conclusion

This study provides the first integrated antioxidant, phytochemical, and *in silico* characterization of *Tetradenia riparia* and *Tetracera poggei* as sources of antioxidant compounds and potential DRBD18 ligands warranting further biological investigation. Both species exhibited significant antioxidant activity, with their extracts giving IC_50_ values below 100 μg/mL. Among the extracts tested, the percolated extracts from *T. poggei* showed the strongest DPPH radical scavenging activity, with an IC_50_ value of 4.141 ± 0.175 μg/mL. UPLC-QTOF-MS identified complementary metabolite profiles including rosmarinic acid, diterpenoids, and flavonoids dominating in *T. riparia*, and galloylated catechin derivatives in *T. poggei*. Molecular docking against DRBD18 identified diterpenoid SP-II and (−)-epicatechin-3-(3-O-methyl)-gallate as potential DRBD18 ligands and candidate inhibitors (RRM1: ΔG = −7.3 kcal/mol), with a consistent structure–activity relationship linking polyphenolic and terpenoid complexity with predicted RRM binding affinity. Taken together, the experimentally demonstrated antioxidant activity and the predicted interaction of the identified compounds with DRBD18 provide a systematic basis for the traditional use of these plants and highlight their potential as sources of lead compounds for the development of plant-derived trypanocidal agents. Experimental validation through *in vitro* and *in vivo* studies remains an essential next step to confirm their activity against *Trypanosoma brucei*.

## Data Availability

The metabolomics data have been deposited to MetaboLights (Yurekten et al., 2024) repository with the study identifier MTBLS15597.
