# Supplementary material for: Bioactive compounds from Tetradenia riparia and Tetracera poggei: antioxidant activity, UPLC-QTOF-MS phytochemical profiling, and in silico targeting of the RNA-binding protein DRBD18 of Trypanosoma brucei
Source: Front Pharmacol. 2026 Sep 22;17:1910102. doi: 10.3389/fphar.2026.1910102 (PMC13639295; doi:10.3389/fphar.2026.1910102)
Supplement: Supplementary file 1 [file Supplementaryfile1.docx]

**Supplementary information**

**Figure S1.** Bubble diagram of protein–ligand interactions illustrating residue-level contacts between epi-gallocatechin-3-O-gallate (RRM1) and catechin-3-O-gallate (RRM2), highlighting hydrogen bonds, π interactions, and interaction distances.

**Figure S2.** Bubble diagram of protein–ligand interactions showing residue-level contacts of diterpenoid SP-II with RRM1 and RRM2, highlighting hydrogen bonds, hydrophobic contacts, and interaction distances.
